# Supplementary material for: Molecular mechanism for the synchronized electrostatic coacervation and co-aggregation of alpha-synuclein and tau
Source: Nat Commun. 2022 Aug 6;13:4586. doi: 10.1038/s41467-022-32350-9 (PMC9357037; doi:10.1038/s41467-022-32350-9)
Supplement: Supplementary file 1 — Supplementary Information [file 41467_2022_32350_MOESM1_ESM.pdf]

Supplementary information for

Molecular Mechanism for the Synchronized Electrostatic Coacervation and Co-Aggregation of Alpha-Synuclein and Tau

Pablo Gracia, David Polanco, Jorge Tarancón-Díez, Ilenia Serra, Maruan Bracci, Javier Oroz, Douglas V. Laurents, Inés García and Nunilo Cremades

Supplementary Methods

Aggregation assays

ThT-monitored aggregation assays were performed as described by Sandberg & Nyström[1]. 50 µM of Tau441, AggDef-Tau or K18 monomer were incubated in PB buffer pH 7.4, 25 mM NaCl, 25 µM ThT, 0.02 % azide, in the presence of 12.5 µM of heparin (Sigma-Aldrich) at 37 °C and under shaking conditions (700 rpm using in situ orbital agitation in the plate reader) until reaction was complete. Non-Binding 96-Well Microplate (µClear®, Black, F-Bottom/Chimney Well), Greiner bio-one North America Inc., USA) were used and the plates were covered with adhesive foil to prevent evaporation. All buffer samples and additive stock solutions were pre-filtered with 0.22 mm filters and the multi-well plates were thoroughly cleaned before use. Kinetic reads were recorded in a FLUOstar plate reader (BMG Labtech, Germany) with excitation at 450 ± 5 nm and emission at 485 ± 5 nm.

Atomic force microscopy

20 µL of Tau441 aggregates resulting from heparin-induced aggregation as described above and αS/Tau441 isolated puncta from 24 h samples as described in Liquid-to-solid phase transition (LSPT) puncta isolation (Methods) were deposited on freshly cleaved Muscovite Mica V-5 (Electron Microscopy Sciences; Hatfield, Pensilvania, USA). Slides were washed three times with more than 5 mL of double distilled water to remove PEG and salts and allowed to dry before imaging acquisition on a Bruker Multimode 8 (Bruker; Billerica, USA) using a FMG01 gold probe (NT-MDT Spectrum Instruments Ltd., Russia) in intermittent-contact mode in air. Images were processed using Gwyddion 2.56 and the width measurements were corrected for the tip shape and size (10 nm).

Supplementary References

1. Sandberg, A. & Nyström, S. Purification and fibrillation of recombinant human amyloid-β, prion protein, and Tau under native conditions. in Amyloid Proteins (eds. Sigurdsson, E. M., Calero, M. & Gasset, M.) 1779, 147–166 (Springer, 2018).

Supplementary Figures

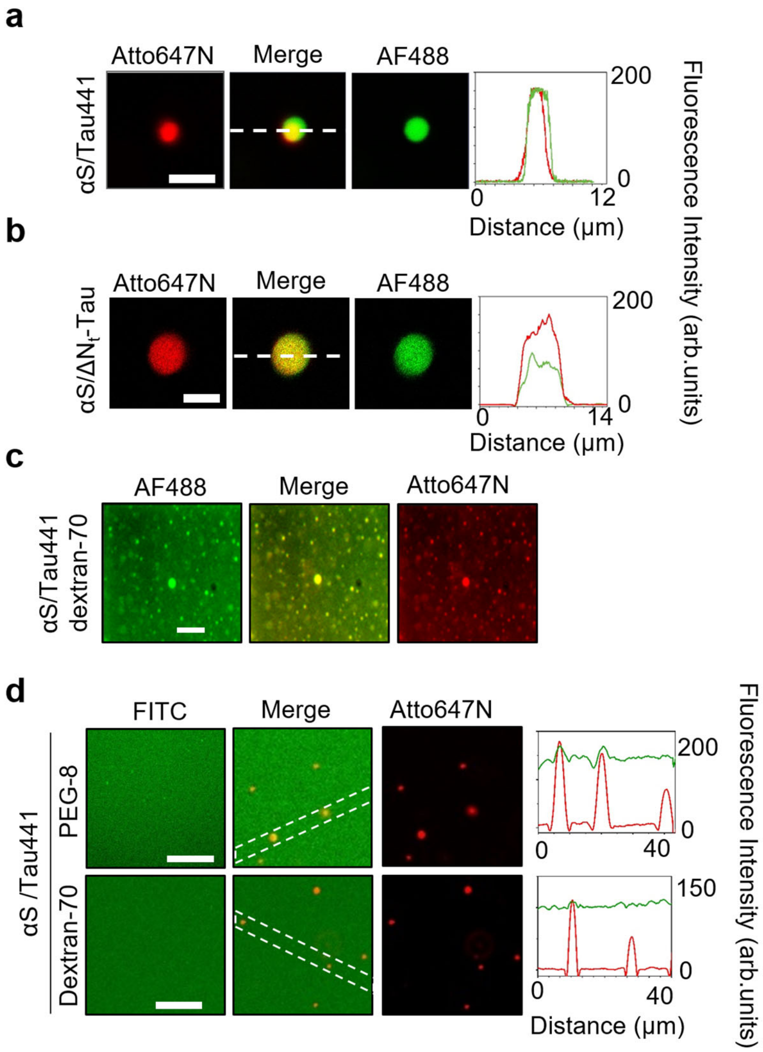

Supplementary Figure 1. Spatial distribution of αS, Tau441, ΔN$_t$-Tau and macromolecular crowders in the coacervate samples. Representative confocal fluorescence (CF) microscopy images of colocalizing αS/Tau441 (**a**, 10 µM each protein) and αS/ΔN$_t$-Tau (**b**, 25 µM each) coacervates. The intensity profiles obtained at the center of the image (shown as discontinuous lines in the center of the merge image) are shown for the fluorescence intensity of αS-AF488 (green) and Tau441- or ΔN$_t$-Tau-Atto647N (red). **c**) Representative WF microscopy images of αS/Tau441 coacervates (25 µM each) in 20 % dextran-70 (w/v). **d**) Representative CF microscopy images of αS/Tau441 LLPS in different crowders. In green, the fluorescence intensity of FITC-labeled crowders (1 % with respect to the total crowder concentration) is shown and, in red, Atto647N-labeled αS (1 µM). For the coacervate samples in the presence of PEG-8, αS and Tau were used at 10 µM each protein and PEG at 15 % (w/v), while for the coacervate samples in the presence of dextran-70, the proteins were used at 25 µM each, and the crowder at 20 % (w/v). The dotted white rectangle indicates the analyzed area used to obtain the fluorescence intensity profiles shown in the right panels. Scale bars are 5 µm in panels **a-c** and 10 µm in panel **d**. Experiments were performed in triplicate with comparable results.

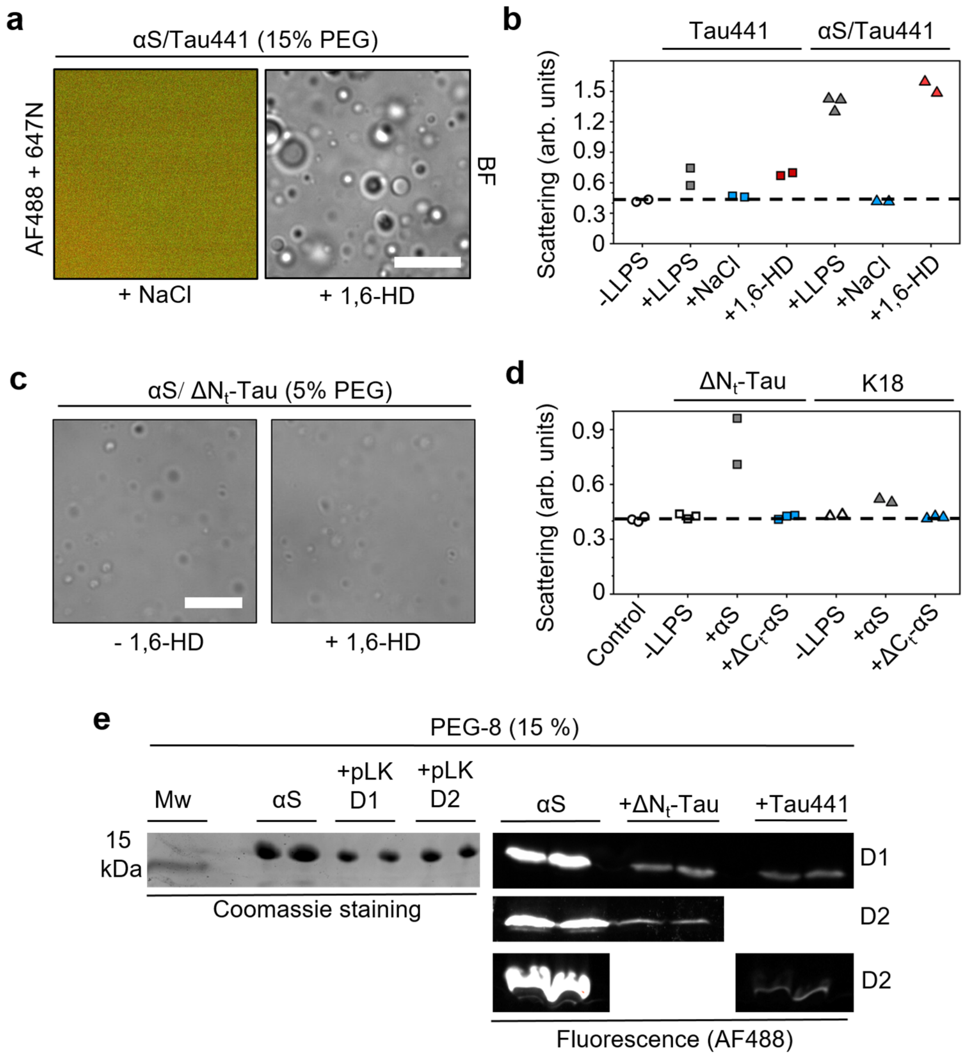

Supplementary Figure 2. Role of electrostatic interactions and quantification of the coacervation of αS with the poly-cations. a) Representative WF (left) and BF (right) microscopy images of the effect of 1 M NaCl and 10 % (v/v) 1,6-Hexanediol (1,6-HD) on αS/Tau441 coacervates (10 µM each protein, 1 µM AF488-αS and Atto647N-Tau441 for WF microscopy). Scale bar = 20 µm. **b**) Light scattering (at 350 nm) of Tau441 and αS/Tau441 coacervate samples (10 µM each protein) in the absence or the presence of 1 M NaCl or 10 % 1,6-HD (N=2-3 sample replicas, as indicated). **c)** Representative WF microscopy images of αS/ΔN$_t$-Tau coacervates (50 µM each protein) formed in the presence of 5 % PEG, in the absence (left) or presence (right) of 10 % (v/v) 1,6-HD. Scale bar is 20 µm. **d**) Light scattering (at 350 nm) of αS or ΔC$_t$-αS with ΔN$_t$-Tau or K18 (50 µM each protein) in LLPS buffer (15 % PEG) (N=2-3 sample replicas, as indicated). Samples without proteins served as control. Samples with only ΔN$_t$-Tau or K18 in LLPS buffer are indicated as –LLPS samples for each protein data set, as no droplets are observed. **e**) Quantification of the fraction of αS in the dispersed phase in the different LLPS systems: 100 µM αS with 1 mM pLK or 100 µM Tau441 or ΔN$_t$-Tau (100% was taken from the samples of αS in the absence of poly-cation in each independent gel). The LLPS samples were centrifuged after 30 min incubation and the fraction of αS remaining in the disperse phase ($f_{\alpha S,d}$) was determined by SDS-PAGE gel analysis. The protein bands were resolved by coomassie staining for the αS/pLK system and by fluorescence for the αS/Tau441 and αS/ΔN$_t$-Tau systems (for both αS and Tau variants quantification —1 µM of fluorescently-labeled proteins— although only αS quantification is shown). D1 and D2 indicate independent duplicate experiments. Each individual gel was

analyzed independently. Source data with the unprocessed gel images are provided as a Source Data file.

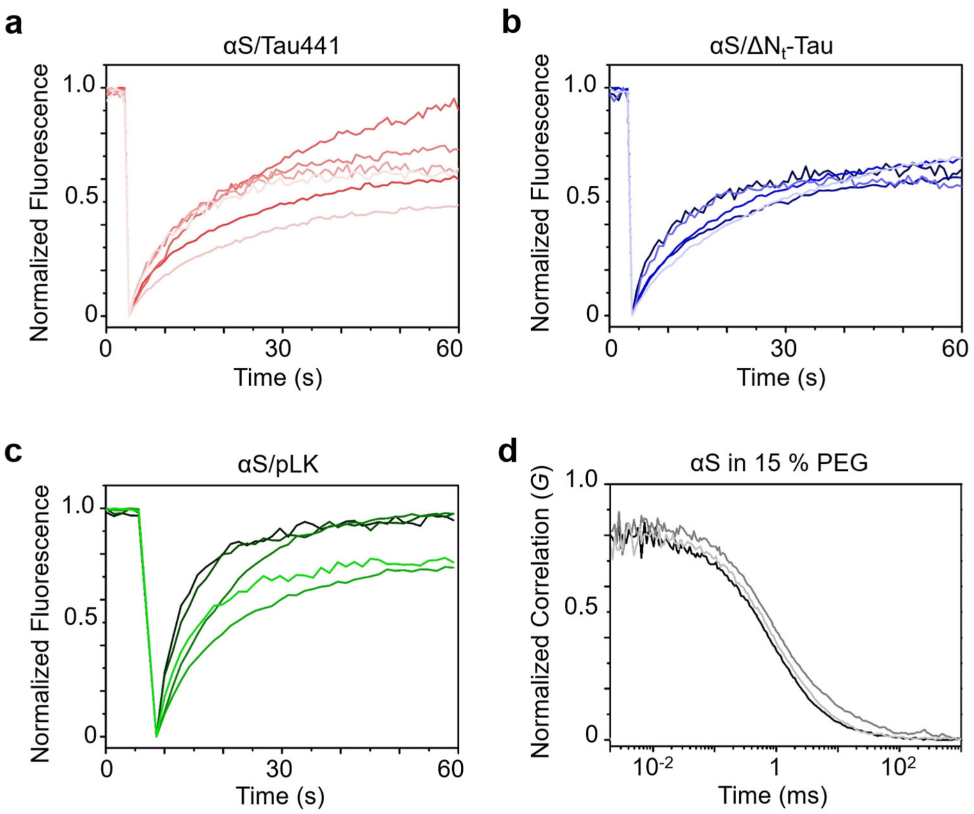

Supplementary Figure 3. Dynamics of αS inside electrostatic complex coacervates by Fluorescence Recovery After Photobleaching (FRAP) and in the dispersed phase by Fluorescence Correlation Spectroscopy (FCS). (**a-c**) Normalized FRAP curves of αS coacervates with Tau441 (**a**), ΔN_t-Tau (**b**) or pLK (**c**). The kinetics of fluorescence recovery of 5 different droplets from 3 independent replicas are shown. (**d**) Normalized FCS curves from a triplicate experiment with 1 nM AF488-αS using the same conditions as in **a-c** but without any poly-cation. Source data are provided as a Source Data file.

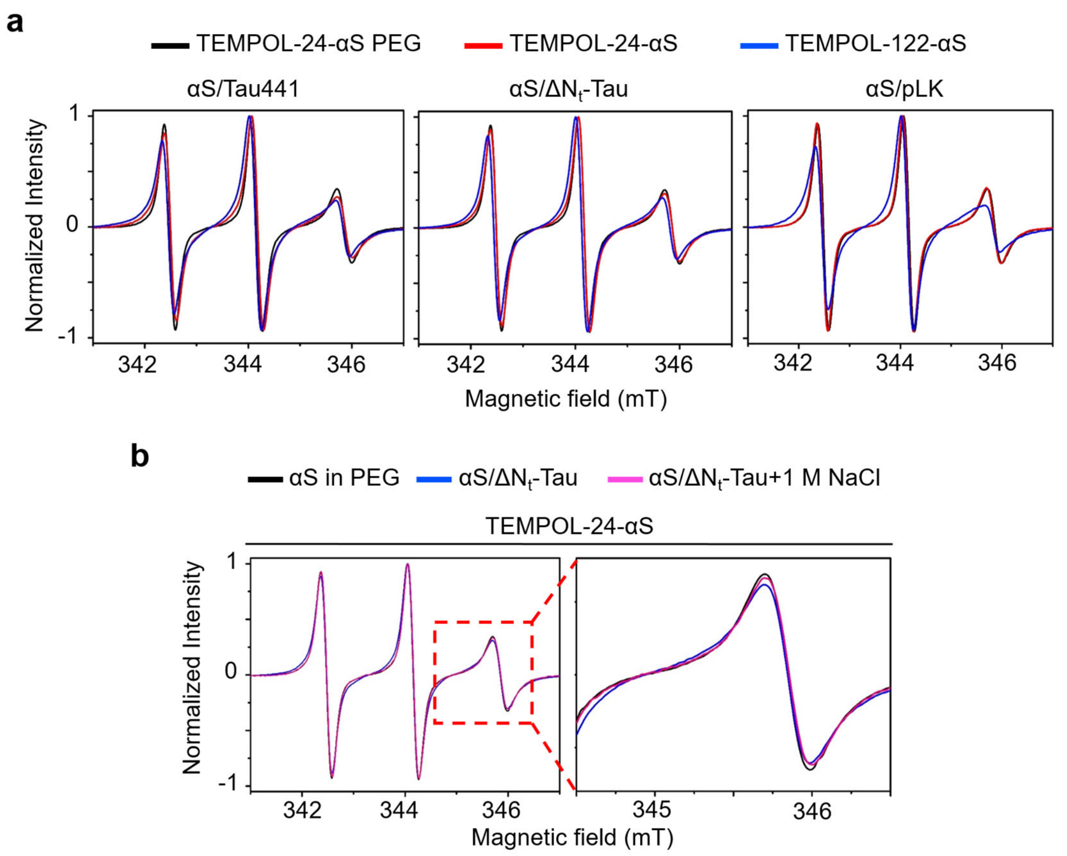

Supplementary Figure 4. Dynamics of αS in electrostatic complex coacervate samples by EPR. a) Normalized CW X-Band EPR spectra of 100 μM TEMPOL-24-αS in 15 % PEG-8 (black) and TEMPOL-24-αS (red) or TEMPOL-122-αS (blue) in the presence of 100 μM Tau441 (left) or ΔN_t-Tau (center) or 1 mM pLK (right). **b**) Normalized CW X-Band EPR spectra of 100 μM TEMPOL-24-αS under LLPS conditions with ΔN_t-Tau before (blue) and after (pink) addition of 1 M NaCl. The spectrum of αS under LLPS conditions but without ΔN_t-Tau is shown in black for comparison. The inset shows a zoom into the high-field band, where the most significant changes occur. Source data are provided as a Source Data file.

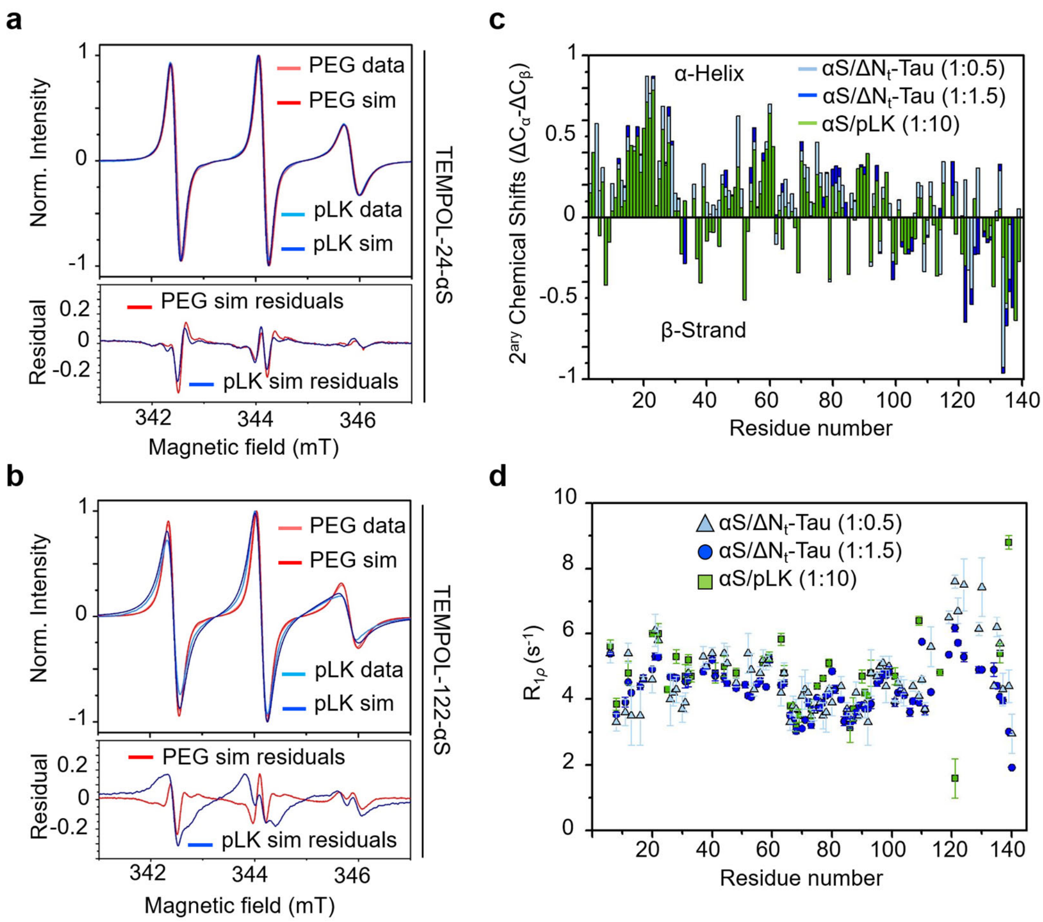

Supplementary Figure 5. Restricted dynamics of the C-terminus of αS upon interaction with poly-cations in electrostatic complex coacervate samples by EPR and NMR. a-b) Normalized CW X-Band EPR spectra and simulations of TEMPOL-24-αS (**a**) and TEMPOL-122-αS (**b**) in 15 % PEG with (blue) or without (red) 10-molar equivalents of pLK. Best simulations are shown in dark blue and dark red for labeled αS with or without pLK, respectively. The EPR signal of the protein in the dispersed phase is consistent with a nitroxide radical in the fast motion regime, characterized by g_{iso} = 2.0055 and axial hyperfine coupling (A = [20 20 104] MHz). For the spectra of TEMPOL-24-αS, a one-component isotropic model with virtually identical correlation times for the sample with and without pLK was obtained (corr. time $6.5 \cdot 10^{-10}$ s). For TEMPOL-122-αS, the sample in absence of pLK could be well simulated to one-component isotropic model with a corr. time $7.5 \cdot 10^{-10}$ s, while two-component isotropic model was needed to simulate the data for the sample with pLK (30% with a corr. time of $7.5 \cdot 10^{-10}$ s and 70% with a slower component with corr. Time $2.0 \cdot 10^{-9}$ s). Residuals were calculated as described in the "Methods" section and are shown for labeled αS with (dark blue) or without (dark red) pLK. **c)** Secondary chemical shifts and (**d**) $R_{1\rho}$ relaxation analysis for 150 μM $^{13}C/^{15}N$-labeled αS in the presence of 1.5 mM pLK (green), 75 μM ΔN_t-Tau (light blue) or 225 μM ΔN_t-Tau (dark blue), respectively. Data and error bars correspond to the $R_{1\rho}$ rates and experimental uncertainties obtained from fitting the intensities of the peaks over the distinct time delays to an exponential function. The data show the reduction of conformational flexibility in the C-terminal region of αS upon interacting with poly-cations. Source data are provided as a Source Data file.

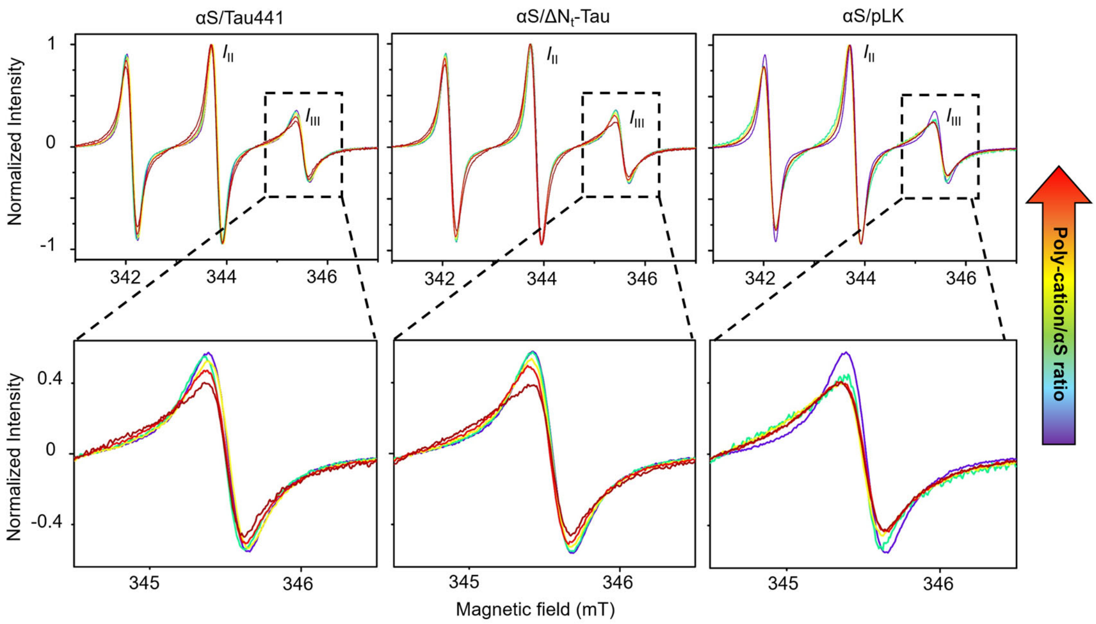

Supplementary Figure 6. EPR binding titration of αS and different poly-cations. Normalized CW X-Band EPR spectra (top) and zooms (bottom) of 50 µM TEMPOL-122-αS in 15 % PEG-8 in the presence of increasing concentrations of Tau441 (left), ΔN_t-Tau (center) or pLK (right). A cold-to-hot color code indicates increasing concentrations of the poly-cation. The second and third bands of the spin probe spectrum, used for the titration analysis (see Fig. 3**e** and Methods for more information) are indicated as I_{II} and I_{III}, respectively, where *I* stands for intensity. Source data are provided as a Source Data file.

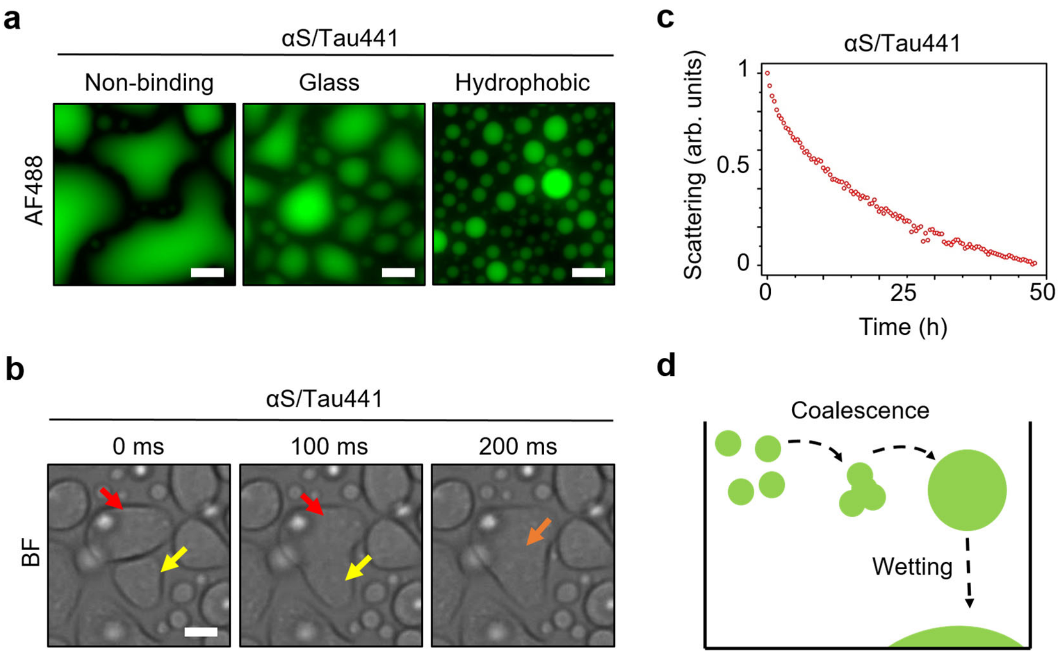

Supplementary Figure 7. Coalescence and wetting properties of αS/Tau441 coacervates. a) Representative WF microscopy images of αS/Tau441 coacervates (25 μM each protein, 1 μM AF488-labeled αS) visualized at the bottom of the plate wells (z = 0 μm) after 24 h incubation using microwell plates with different material coatings as indicated. Both non-binding plates (PEG-based coated microwell plates typically used as non-binding plates for hydrophobic protein samples) and glass plates have hydrophilic coatings. As hydrophobic material, non-treated 96-well polystyrene microplates were used. **b**) A fusion event of two αS/Tau441 rafts as observed by BF microscopy is shown. Scale bars are 20 μm. **c**) Light scattering signal (at 350 nm) of an αS/Tau441 coacervate sample over time, indicating the time scale for the deposition and wetting of the droplets on the bottom of the sample. Source data are provided as a Source Data file. **d**) Schematic of the coalescence and surface wetting of αS/Tau441 coacervates.

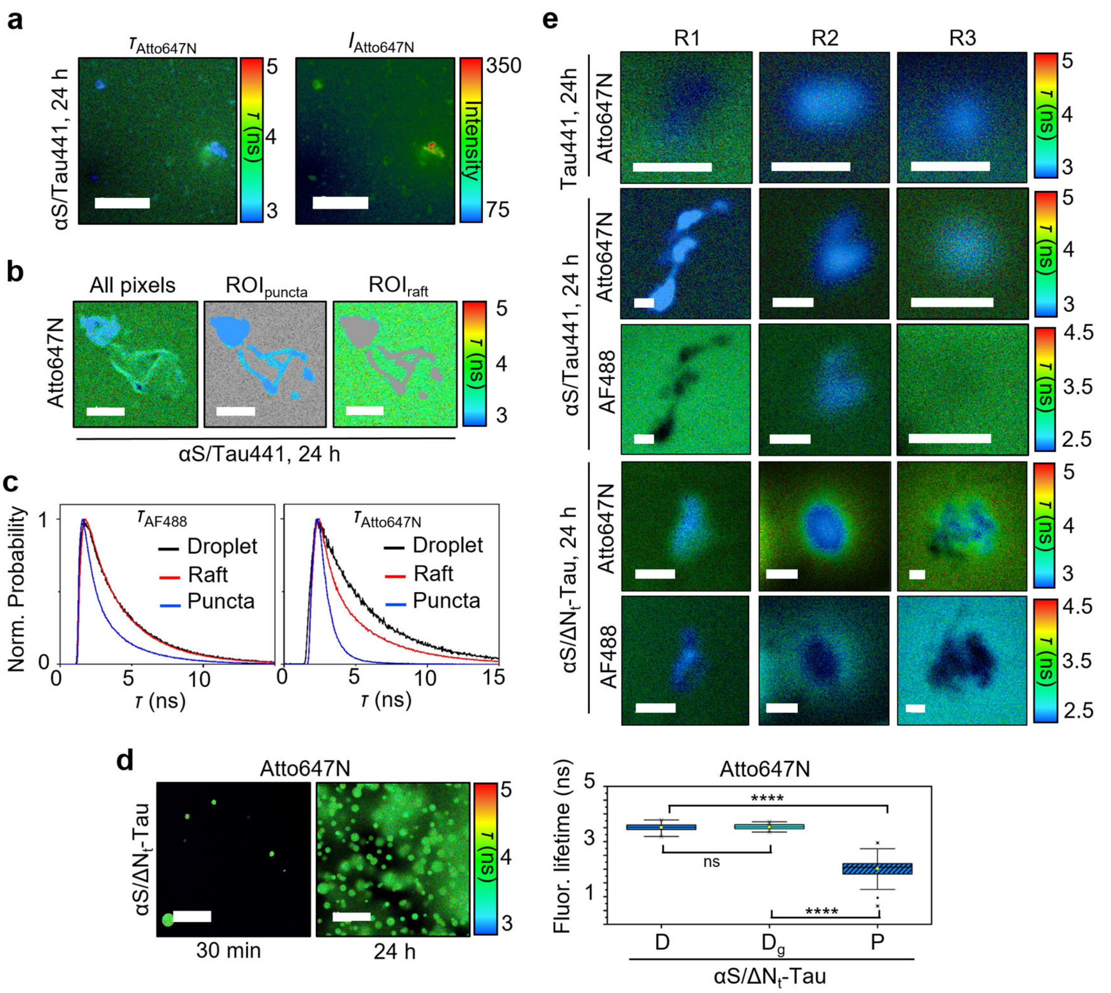

Supplementary Figure 8. Fluorescence lifetime imaging microscopy (FLIM) analysis of the LSPT in αS/Tau441 coacervates. a) Representative fluorescence lifetime (left) and intensity (right) color-coded images of αS/Tau441 coacervates after 24 h incubation and deposited on the bottom of the well (25 μM each protein, 1 μM AF488-αS and Atto647N-Tau441). The images show the same microscopy field with a large raft composed of both proteins occupying the entire field (shown in green in both images – note that the background intensity is ~ 20 counts) in which several puncta are visualized as a condensation of proteins (higher intensity values, visualized as brighter green-yellow-red spots). This correlates with lower fluorescence lifetimes (due to condensation-induced self-quenching) visualized as blue spots that coincide with the more intense spots. Data is shown for the Atto647N channel. Scale bars = 10 μm. **b-c**) Analytical approach for species-specific lifetime analysis. A representative lifetime color-coded image of αS/Tau441 coacervates after 24 h incubation is shown in **b**, and the selection of regions of interest (ROIs) containing species-specific lifetimes (ROI_{puncta}, ROI_{raft}) are selected from the overall image (ROI_{all}). Data are shown for the Atto647N channel. Scale bars are 5 μm. **c**) Representative normalized lifetime decays from species-specific ROIs for the AF488 (left) and Atto647N (right) channels. Source data are provided as a Source Data file. **d**) Representative lifetime color-coded FLIM images (left panels) of αS/ΔN$_t$-Tau droplets at initial times and after 24 h incubation and deposited on the bottom of the well (25 μM each protein, 1 μM AF488-αS and Atto647N-ΔN$_t$-Tau; scale bar = 20 μm) and their pixel-wise lifetime analysis (right, box plot). 24h-incubated gelated droplets (D_g) are shown in light turquoise blue (N = 10). Early droplets (D) and puncta (P) are the same as in Fig. 6c but are shown here for comparison. Source data are provided as a Source Data file. Mean and median values are

shown as yellow squares and black lines within the boxes, respectively. Lower and upper box limits indicate the first and third quartile, respectively, while minimum and maximum values within 1.5 x interquartile range (IQR) are shown as whiskers. Outliers are shown as black diamonds. The statistical significance between pairs of distributions was determined with a two sample t-test assuming unequal variances. No significant differences were found for droplets that gelated after 24-h incubation (D_g). The *p*-value from a two-tailed t-test is shown as stars for each compared pair of data (* *p*-value > 0.01, ** *p*-value > 0.001, *** *p*-value > 0.0001, **** *p*-value > 0.00001, *ns* means not significant (*p*-value > 0.05). Precise p-values are provided in Supp. Table 1. **e**) Lifetime color-coded FLIM images showing the heterogeneity of size, shape and fluorescence lifetime in puncta from Tau441, αS/Tau441 and αS/ΔN_t-Tau. For αS/Tau441 and αS/ΔN_t-Tau puncta the variability in αS content is also evident. Scale bar = 1 μm

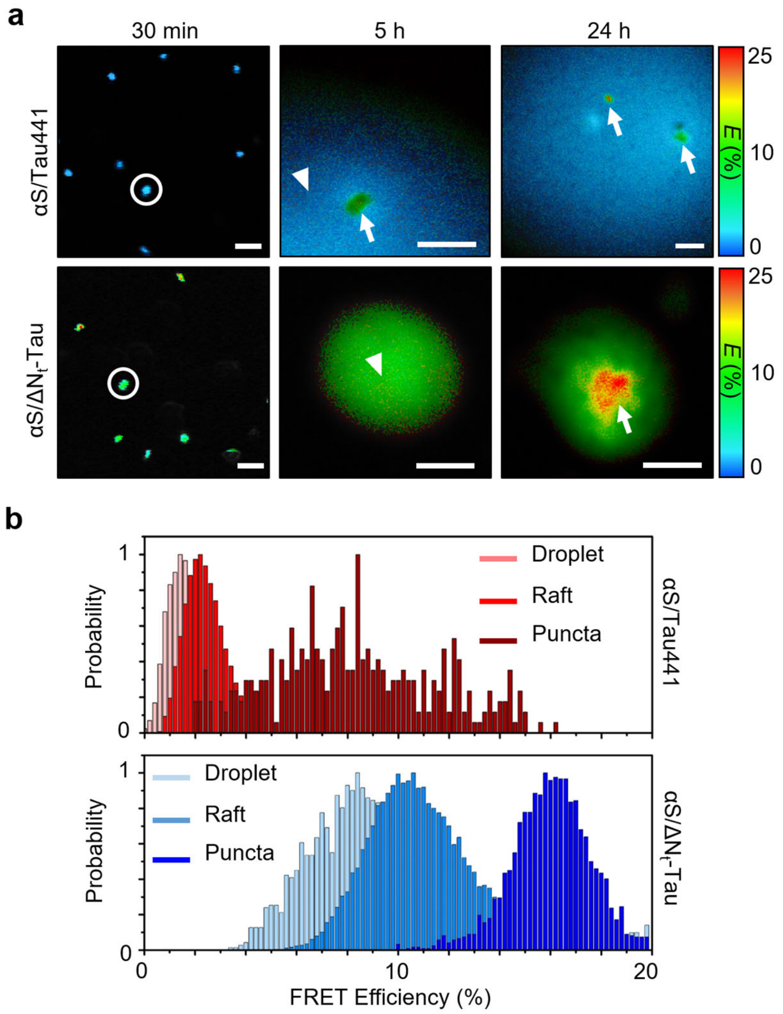

Supplementary Figure 9. Förster Resonance Energy Transfer (FRET) analysis of the LSPT in αS electrostatic coacervates. a) Representative FRET efficiency (*E*) color-coded FRET images of αS/Tau441 (top) and αS/ΔN_t-Tau (bottom) coacervation at *t* = 30 min (left), 5 h (center) and 24 h (right). Higher, "red-shifted", *E* values in puncta (white arrows) with respect to the droplets (white circles) and rafts (white arrowheads) indicate LSPT. Scale bars are 5 μm for the left images and 2 μm for the center and right images. **b**) Normalized FRET efficiency histograms for the different protein structures observed (droplets, rafts and puncta) in αS/Tau441 (top) and αS/ΔN_t-Tau (bottom) LLPS and LSPT processes. Each histogram is composed of a triplicate experiment with 2 analyzed fields per sample. Source data are provided as a Source Data file.

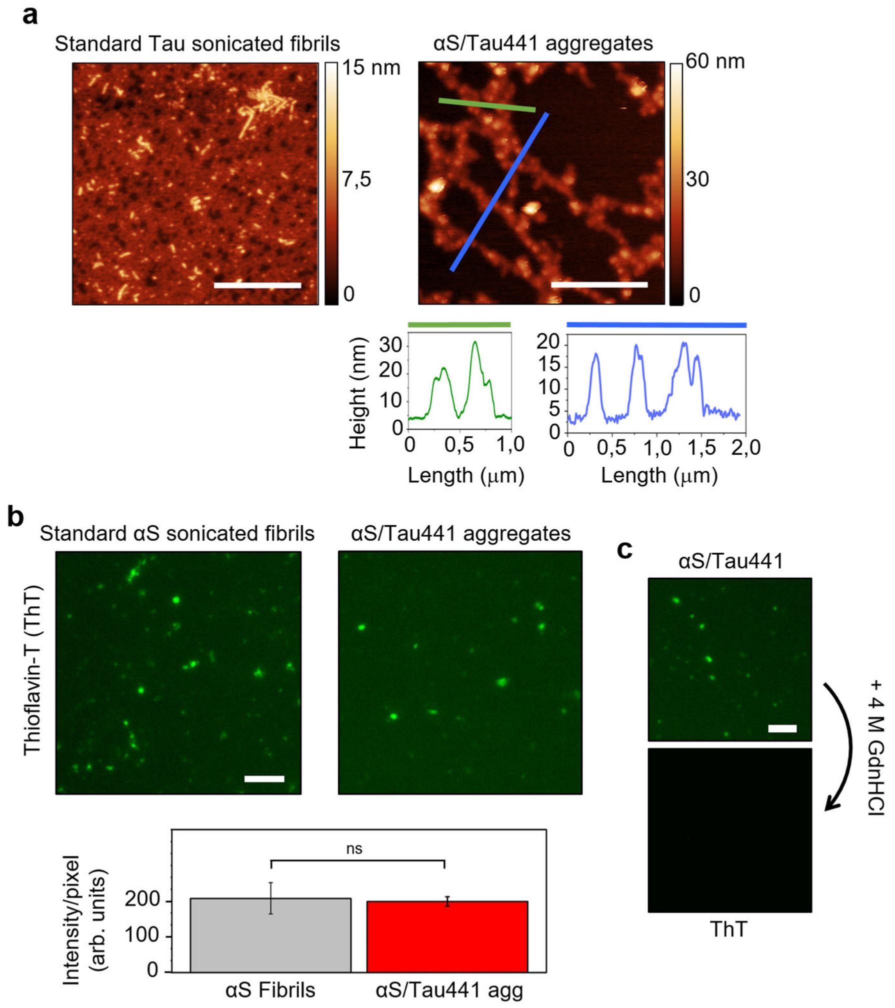

Supplementary Figure 10. Analysis of the isolated αS/Tau441 aggregates generated inside the coacervates. a) Representative AFM image of the isolated αS/Tau441 aggregates and the analysis of the corresponding aggregate heights. A typical image of the standard Tau sonicated amyloid fibrils is shown as comparison. **b**) Representative WF microscopy image of a ThT staining (1:2 ThT:αS) of sonicated canonical αS fibrils (left) and isolated αS/Tau441 aggregates (right), both in buffer without PEG but with 1 M NaCl. The treatment with high salt concentration dissolves the rafts and any possible droplets that remained suspended in the solution. The puncta, however, are insensitive to this treatment and remain stable, indicating that the main interactions that stabilize the protein structures in the coacervates versus the puncta are different. A control sample with sonicated αS fibrils treated in the same way as the puncta (fibrils were deposited on the bottom of the wells once formed and quantified and then resuspended in the same buffer with 1 M NaCl as for the puncta) is shown on the left panel. The statistical analysis of the mean particle intensity of the αS/Tau441 aggregates formed inside the protein liquid coacervates by LSPT (red bar), as compared to the typical in vitro-generated αS fibrils (gray bar) is shown below the WF images (N = 90 for the αS fibrils and N = 80 for the LLPS-derived αS/Tau441 aggregates). The analysis was performed from data obtained from 3 independent samples with 3 analyzed fields per sample that contained at least 20 particles per field (see Supplementary Methods for a detail description of the analysis). Source data are provided as a Source Data file. The mean and standard deviation

are shown. The statistical significance between pairs of distributions was determined with a two sample t-test assuming unequal variances, but no significant (ns) difference was obtained. **c**) WF microscopy images of isolated αS/Tau441 aggregates before (top) and after (bottom) addition of 4 M GdnHCl. Scale bars in panels b) and c) are 40 μm.

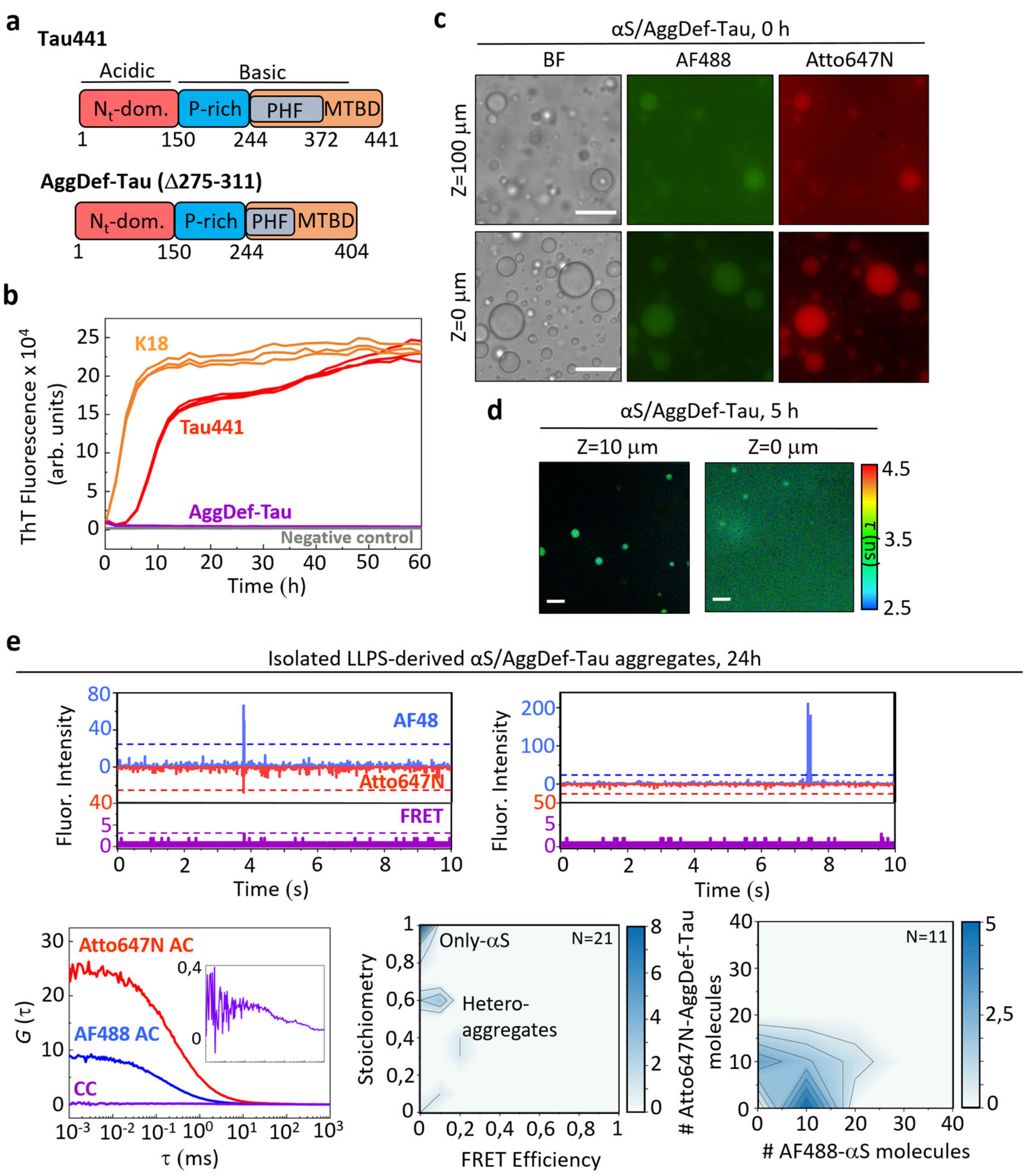

Supplementary Figure 11. LLPS and LSPT of αS and an amyloid aggregation deficient Tau variant. a) Schematic of the different protein regions of Tau and the amyloid aggregation deficient Tau variant, AggDef-Tau, where the region of full-length Tau between residues 275 and 311 was removed. **b)** Aggregation kinetics of Tau441, K18 and the AggDef-Tau variants under typical in vitro Tau amyloid aggregation conditions (50 µM of each Tau variant in PBS buffer, 0.02% azide, in the presence of 12.5 µM heparin and 25 µM ThT). Source data are provided as a Source Data file. **c)** Brightfield (BF, left image) and widefield fluorescence (WF, center and right images) microscopy images of αS/AggDef-Tau coacervates (50 µM each protein, 1 µM AF488-labeled αS and Atto647N-labeled Tau variant, in LLPS buffer) at 0 h of incubation. Scale bar = 20 µm for all the images. **d)** FLIM images of analogous coacervate samples at 5 h of incubation (suspended droplets in the left panel, a raft covering the image in the right panel). The lifetime shown corresponds to Atto647N-AggDef-Tau. Scale bar = 10 µm for both images. **e)** Single-molecule fluorescence burst analysis of isolated aggregates generated inside αS/AggDef-Tau electrostatic coacervates. Two representative

single-molecule fluorescence time traces are shown at the top, where the bursts of aggregates are evident as the only bursts whose intensities are above the established thresholds. These bursts are absent in monomer-only samples (not shown). An example of FCS/FCCS analysis of the isolated aggregates generated inside the αS/AggDef-Tau coacervates is shown at the bottom left panel, where very few aggregates were detected (almost no cross-correlation curve, CC in violet, and the auto-correlation curves, AC in blue and red, showed mainly particles diffusing as monomers). The stoichiometry, S, vs FRET efficiency plot of the few aggregates detected is shown at the bottom-center panel (the color scale reflects occurrence). Most of the aggregates detected contained mainly αS molecules. For the very few hetero-aggregates detected, the estimated number of Atto647N-AggDef-Tau and AF488-αS molecules per aggregate is shown in the bottom-right panel (the color scale reflects occurrence). Source data are provided as a Source Data file.

Supplementary Tables

Supp. Table 1. P-values for the statistical analysis

Figure	Variable	Sample 1	Sample 2	p-value (two tails)	Significance
Figure 3c	Diffusion coef. (FRAP)	*aS/Tau441*	*aS/DNt-Tau*	0,026387529	*
	Diffusion coef. (FRAP)	*aS/Tau441*	*aS/pLK*	0,001146886	**
	Diffusion coef. (FRAP)	*aS/DNt-Tau*	*aS/pLK*	0,001583742	**
Figure 6c	τ_Atto647N (FLIM)	*aS/Tau441 Droplets*	*aS/Tau441 Rafts*	0,00037838	***
	τ_Atto647N (FLIM)	*aS/Tau441 Droplets*	*aS/Tau441 Puncta*	2,39117E-28	****
	τ_Atto647N (FLIM)	*aS/Tau441 Rafts*	*aS/Tau441 Puncta*	7,31582E-24	****
	τ_Atto647N (FLIM)	*aS/dNt-Tau Droplets*	*aS/dNt-Tau Rafts*	8,65213E-06	****
	τ_Atto647N (FLIM)	*aS/dNt-Tau Droplets*	*aS/dNt-Tau Puncta*	1,36169E-16	****
	τ_Atto647N (FLIM)	*aS/dNt-Tau Rafts*	*aS/dNt-Tau Puncta*	1,03263E-13	****
	τ_Atto647N (FLIM)	*aS/Tau441 Droplets*	*aS/dNt-Tau Droplets*	0,024771104	ns
	τ_Atto647N (FLIM)	*aS/Tau441 Rafts*	*aS/dNt-Tau Rafts*	0,154397359	ns
	τ_Atto647N (FLIM)	*aS/Tau441 Puncta*	*aS/dNt-Tau Puncta*	8,44311E-05	****
	τ_AF488 (FLIM)	*aS/Tau441 Droplets*	*aS/Tau441 Rafts*	0,940650246	ns
	τ_AF488 (FLIM)	*aS/Tau441 Droplets*	*aS/Tau441 Puncta*	0,000434654	***
	τ_AF488 (FLIM)	*aS/Tau441 Rafts*	*aS/Tau441 Puncta*	0,000438693	***
	τ_AF488 (FLIM)	*aS/dNt-Tau Droplets*	*aS/dNt-Tau Rafts*	0,000981013	***
	τ_AF488 (FLIM)	*aS/dNt-Tau Droplets*	*aS/dNt-Tau Puncta*	4,32225E-06	****
	τ_AF488 (FLIM)	*aS/dNt-Tau Rafts*	*aS/dNt-Tau Puncta*	0,000562987	***
	τ_AF488 (FLIM)	*aS/Tau441 Droplets*	*aS/dNt-Tau Droplets*	0,13058537	ns
	τ_AF488 (FLIM)	*aS/Tau441 Rafts*	*aS/dNt-Tau Rafts*	4,11896E-05	****
	τ_AF488 (FLIM)	*aS/Tau441 Puncta*	*aS/dNt-Tau Puncta*	0,018219485	*
Supp. Fig. 10b	ThT intensity/pixel	*aS sonicated fibrils*	*aS/Tau441 agg*	0,474388524	ns
